# Electrospun Scaffolds and Induced Pluripotent Stem Cell-Derived Cardiomyocytes for Cardiac Tissue Engineering Applications

**DOI:** 10.3390/bioengineering7030105

**Published:** 2020-09-06

**Authors:** Taylor Cook Suh, Alaowei Y. Amanah, Jessica M. Gluck

**Affiliations:** Textile Engineering, Chemistry and Science Department, Wilson College of Textiles, NC State University, Raleigh, NC 27695, USA; tacook3@ncsu.edu (T.C.S.); ayamanah@ncsu.edu (A.Y.A.)

**Keywords:** tissue engineering, cardiac tissue engineering, engineered heart tissue, electrospinning, scaffolds, cardiomyocytes, induced pluripotent stem cells

## Abstract

Tissue engineering (TE) combines cells, scaffolds, and growth factors to assemble functional tissues for repair or replacement of tissues and organs. Cardiac TE is focused on developing cardiac cells, tissues, and structures—most notably the heart. This review presents the requirements, challenges, and research surrounding electrospun scaffolds and induced pluripotent stem cell (iPSC)-derived cardiomyocytes (CMs) towards applications to TE hearts. Electrospinning is an attractive fabrication method for cardiac TE scaffolds because it produces fibers that demonstrate the optimal potential for mimicking the complex structure of the cardiac extracellular matrix (ECM). iPSCs theoretically offer the capacity to generate limitless numbers of CMs for use in TE hearts, however these iPSC-CMs are electrophysiologically, morphologically, mechanically, and metabolically immature compared to adult CMs. This presents a functional limitation to their use in cardiac TE, and research aiming to address this limitation is presented in this review.

## 1. Introduction

Tissue engineering (TE) is a field that combines cells, scaffolds, and biologically active signaling molecules, to assemble functional tissues, with the ultimate goal of repairing and replacing tissues and organs. These three components are often referred to as the three “pillars” of TE. Cells used in cardiac TE approaches can be autologous, allogenic, cell lines, primary lines, or progenitor cells. Scaffolds refer to the materials upon which cells are placed to promote growth and proliferation. These scaffolds aim for high biocompatibility and biomimicry, i.e., mimicking the in vivo environment. Many methods are used for the fabrication of TE scaffolds. Electrospinning is an attractive fabrication method because the fibers it produces demonstrate high potential for mimicking the complex structure of the cardiac ECM. Scaffolds can also offer varying additional functions through structural, mechanical, and/or electrical properties. Biologically active signaling molecules include growth factors and cytokines, which are a diverse group of components included to recreate the in vivo environment that facilitates differentiation, growth, and/or functional development of cells. These growth factors and cytokines can be biological, mechanical, chemical, electrical, or any combination thereof.

Cardiac TE is the application of TE to the development of cardiac cells, tissues, and structures. This is a critical area of research as it proposes an alternative to heart transplants, which, if lifestyle changes and pharmaceutical intervention prove insufficient, are the final option for the treatment for most cardiovascular diseases. These cardiovascular diseases are the leading cause of heart failure and mortality in the United States [1]. In 2015, they accounted for 23.4% of all deaths in the U.S., killing 630,000 people [2]. An American dies from cardiac causes approximately every 37 s [3].

Heart transplants are inadequate treatments for cardiovascular disease and heart failure due to massive shortages in donor organs and long-term challenges involving the chronic rejection of the donated organs by the donor’s immune system [1]. In the U.S. in 2018, there were 2596 patients on the waitlist for a heart transplant, and only 3440 heart transplants were performed [4].

Cardiac TE has specific considerations and constraints that relate to the three components of TE previously described. A viable cardiac TE construct must support cardiomyocytes (CMs) organized into functional tissue capable of generating 2–4 mN/mm^2^ of force during contraction and propagating electrical signals at the rate of approximately 25 cm/s [5]. Any non-cell component of the construct must be biocompatible to ensure there is no toxic or host/foreign body response by the immune system due to the TE construct’s implantation.

Several central issues regarding cardiac TE must be addressed before the field can provide a feasible alternative to current cardiac disease and heart failure treatments. These issues or ‘bottlenecks,’ were identified by Bruyneel et al. in 2018 as scale-up, viability and vascularization, immunogenicity and rejection, maturity, and electromechanical integration [6].

The human myocardium contains approximately 10^9^ cells, approximately ⅓ of which are CMs [6]. Cardiac non-myocytes account for the remainder of the population of cells in the human myocardium. Recent findings suggest that approximately 60% of the non-myocyte cells are endothelial cells (ECs), 5–10% are hematopoietic-derived cells, and less than 20% are fibroblasts [7]. All cell types interact with each other post-cardiac injury to maintain the heart’s integrity and pumping effectiveness. The CMs are responsible for generating the heart’s contractile force, while the non-myocytes play an essential role the in homeostatic regulation of the heart. For example, the ECs promote neovascularization and CM organization post-ischemic injury, the fibroblasts synthesize the ECM, and the hematopoietic-derived cells function as effectors of the immune response [8].

The issue of immunogenicity and the possible rejection of engineered cardiac tissue by the body in response to implantation could potentially be eliminated through the use of autologous iPSC (induced pluripotent stem cell)-derived CMs—such as those explored in Zhang et al.’s research published in 2009 [9]. iPSC-CMs are CMs derived from iPSCs and will be discussed more thoroughly later in this review. “Autologously derived” refers to the iPSC-CMs being autologous, i.e., obtained from the same individual that will receive the tissue-engineered construct.

The physiological density required to facilitate adequate contractile force is approximately 10^8^ cells/cm^3^ [5]. The level of scale-up required to generate this volume and density of cells has posed challenges for the development of engineered cardiac tissue [6]. The use of induced pluripotent stem cell-CMs (iPSC-CMs) holds promise in addressing this issue, as iPSC-CMs can be produced in virtually limitless quantities [10]. However, iPSC-CMs introduce limitations because they are immature relative to adult CMs. Huang et al. (2018) considered the maturity of cells in another “bottleneck” in cardiovascular TE [6]. The reasons this immaturity is an issue and research aiming to address it are discussed later in this review.

Another roadblock to developing viable cardiac TE constructs is the high oxygen demand of CMs. At rest and normalized per gram, the myocardium consumes 20× more oxygen than skeletal muscle [11]. This high oxygen demand functionally limits engineered myocardial tissue thickness to approximately 200 μm [5] or even as thin as <100 μm [12]. For context, a single adult human CM is approximately 100 μm long and 10–25 μm in diameter [13]—meaning engineered myocardial tissue can be limited to the thickness of a single CM cell. Several methods have been developed aiming to overcome this challenge, including assembly of cell-cultured monolayers into stacks or “sheets” to create cardiac patches [14], culturing cells on nanofibers imprinted onto solid substrates with specific distances between them, and suspending nanofibers seeded with cells in three-dimensional space [12]. The high oxygen demand of CMs relates to the “bottleneck” issue of viability and vascularization [6].

The “bottleneck” of electromechanical integration refers to the need to successfully integrate engineered myocardial tissue into the host myocardium without inducing re-entry arrhythmia or block due to differences between engineered and native tissues in the heterogeneity of cellular organization [15]. These re-entry arrhythmias and blockages can cause irregular heartbeats, such as tachycardia and bradycardia. Therefore, it is vital to successfully integrate the cardiac TE construct into the native myocardium to prevent them from occurring. This can be achieved by improving electrical communication between engineered tissue and native tissue [10].

## 2. Tissue Engineered Scaffolds

The scaffold component of TE refers to the materials upon which cells—usually stem cells—are seeded to maintain their form and to develop into functional tissue. A TE scaffold aims to act as the ECM, interacting with the seeded cells which are forming de novo tissue [16]. The most important criteria for these scaffolds are biomimicry (i.e., biologically mimicking natural in vivo environments) and biocompatibility.

Functionally, TE scaffolds aim to fulfill the following demands: high biocompatibility; appropriate fiber diameter and pore size for facilitating cell attachment and migration; sufficient surface area and appropriate surface chemistry to encourage cell adhesion, growth, migration, and differentiation; robust mechanical properties; and biodegradation rate similar to regeneration rate of the tissue being engineered [12,16,17,18,19,20,21,22,23,24,25,26]. For cardiac TE, the scaffold must also permit cells to perform a contractile function with no impedance [12]. These factors combine to achieve the ultimate goal of providing a microenvironment for native cells which structurally, mechanically, electrically, and biochemically mimics native tissue [27]. To propagate the electrical signals of the heart, the tissue must be capable of generating contractile forces of 2–4 mN/mm^2^ and propagating electrical signals at ~25 cm/s [5]. A viable cardiac TE scaffold must facilitate this.

### 2.1. Polymers Used for Scaffold Fabrication

The primary basis of biocompatibility is the material from which the scaffold is made. TE scaffolds can be fabricated from synthetic, natural, or a blend/combination of the two materials. In a qualitative review of 72 TE research papers conducted by Jafari et al. in 2015 [16], the most commonly used natural-based scaffolds were of chitosan, collagen, elastin, gelatin, alginate, and silk origin. The most commonly used synthetic-based scaffolds were fabricated from polylactic acid (PLA), polylactic-co-glycolide (PLGA), and polycaprolactone (PCL). Natural polymers offer the advantages of higher abundance and inherent resemblance of components present in ECM. In contrast, synthetic polymers provide the advantages of accommodating chemical modification and possessing high tunability, i.e., offering high versatility and the ability to customize them for specific needs [16,18]. These are two different approaches to biocompatibility. In scaffolds from natural materials, the biocompatibility is intrinsic because the scaffolds typically comprise components which are already present in the body. In scaffolds derived from synthetic materials, biocompatibility comes from ability to customize the scaffold, through fabrication and post-processing methods, to achieve desired characteristics such as certain fiber diameter, pore size, porosity, surface chemistry, etc.

Synthetic polymers PLA, PLGA, and PCL have been shown through biomedical research to offer high biocompatibility. PLA has high biocompatibility partially because it degrades into lactic acid or carbon dioxide and water, all of which are naturally occurring in mammals and metabolically innocuous. These products are harmlessly metabolized intracellularly or harmlessly excreted through urination or respiration processes [19,20]. The degradation of PLA has an approximately 30-week median half-life, but this can be modified to fit certain applications. This demonstrates the previously discussed advantage of tunability in synthetic polymers. For these reasons, foreign body responses to PLA in biomedical and TE applications are extremely rare [19].

PLGA has a chemical composition that closely resembles that of the mineral phase of the bone composition. For this reason, PLGA offers high biocompatibility and is commonly used for bone TE applications [21]. PLGA is a copolymer of PLA and poly(glycolic acid) (PGA) and therefore exhibits a degradation profile in between that of PLA and PGA. It has a relatively fast biodegradation rate, on the scale of weeks to months [22].

PCL offers biocompatibility, although lower than that of PLA or PLGA. It degrades by hydrolysis into ester linkages, which are largely metabolically innocuous and non-toxic [23,24]. PCL has a longer degradation rate and profile, in the range of months to years and some cases multiple years [23,28]. As with other synthetic polymers, this biodegradation timeline is tunable to a certain degree. PCL is widely used in TE and cardiac TE applications, as well as for drug delivery systems and wound healing applications, and as biodegradable stents [24].

### 2.2. Biocompatibility of Scaffolds

The biocompatibility of a TE scaffold is of utmost importance because it plays the greatest role in determining the host response of the human body to the implanted TE construct. If biocompatibility is inadequate, it will trigger immune responses such as acute inflammation, chronic inflammation, granulation tissue formation, foreign body reaction, and tissue encapsulation [25]. Acute inflammation refers to the influx of neutrophils into the implant site, where they interact with proteins adsorbed onto the TE construct’s surface. This process leads to the TE construct eliciting phagocytosis from neutrophils (and later macrophages), or frustrated phagocytosis, wherein microbicidal contents are extracellularly released onto the surface of the scaffold. This release may cause erosion of the scaffold and lead to its failure [26]. The inflammation progresses to a chronic phase when activated macrophages present at the site of construct implantation. If this presence continues, chronic inflammation progresses into the tissue granulation phase, wherein new ECM and growth of vasculature into the site through the process of angiogenesis are conspicuous. This can further progress into a foreign body reaction, involving macrophages and foreign body giant cells [29]. In the final stage of host response, tissue encapsulation will occur, wherein the TE construct is isolated from surrounding healthy tissue via the formation of a dense layer of collagenous connective tissue which entirely encapsulates it [30]. This is an unequivocally undesirable and potentially fatal outcome for the implantation of a TE construct [25,29,31]. A TE scaffold with appropriate biocompatibility will avoid all of these issues, and provide a structure to which cells will adhere to, function normally within, differentiate on, migrate onto the surface of, and eventually lay down the new matrix and de novo tissue formation onto [27,31].

To address the concerns of an immune response to cardiac TE scaffolds, researchers are working on selecting iPSC-CMs that can escape recognition by the immune system, and modifying TE scaffolds that modulate inflammation [6,32]. As noted earlier, the use of autologous iPSCs can avoid rejection of implanted cardiac TE scaffolds. Still, this option remains clinically infeasible due to high associated financial costs, extensive regulatory approval, and prolonged cell culture period to generate personalized constructs [33]. As a result, utilizing human leukocyte antigen (HLA)-matched iPSCs tissue banks for cardiac TE is a potential solution that can address that issue. However, this remains an elusive and challenging option because it requires a concerted global effort to include HLA-matched tissues for a genetically diverse human population [34].

Modification of TE scaffolds to gain higher biocompatibility that can suppress the host response to TE construct implants offers a more feasible option. One method of achieving higher biocompatibility is to use biological or natural-based scaffolds [16]. Another approach involves modifying the surfaces of synthetic scaffolds. A review by Katti, Vasita, and Shanmugam published in 2008 [31] explored surface modifications applied to polymeric scaffolds to increase biocompatibility and provide a delivery vehicle for the proteins necessary for cell attachment and favorable cell interactions. Biomaterial surfaces, which refer to the TE scaffold’s surface, play a vital role in determining the success of the TE construct because it is the location of interaction between native tissue and the implanted biomaterial of the scaffold.

Consequently, employing different strategies that modify the surfaces of cardiac TE scaffolds can attenuate its interaction with immune cells leading to a less pronounced immune response. For example, decreasing hydrophobicity of the surface leads to decreased monocyte adhesion and foreign giant body cell formation, while increasing hydrophilicity limits the maturation of dendritic cells [32]. Additionally, modifying other surface parameters such as the charge, morphology, free energy, and chemical structures can further modulate the immune response and increase favorable interactions between the TE scaffolds and the cells seeded onto it. These parameters influence cell interaction in various ways. The surface charge properties refer to static effects between positively and negatively charged surfaces that favor cell adhesion; surface morphology (rough surfaces favor cell adhesion and proliferation); surface free energy (high surface free energy favors cell adhesion and spreading); and surface chemical structures (presence of amino-, hydroxy-, carboxy-, sulfonic-, and acyl- groups favor cell adhesion and growth).

Surface modifications to acquire desirable characteristics concerning these properties include physical modification, chemical modification, modification through plasma treatment, photochemical modification, modification through composites and graft formations, and modification of polymeric biomaterial surfaces for protein immobilization, modifications with respect to physical adsorption, radiation-mediated modifications, and protein modification [31].

From a biochemical perspective, the promotion of certain signaling pathways with cell/scaffold constructs has proven effective in facilitating stem cells’ survival, proliferation, differentiation, and formation into functional tissue. The specific combination of the growth factors vascular endothelial growth factor (VEGF), basic fibroblast growth factor (b-FGF), platelet-derived growth factor (PDGF-BB), and insulin-like growth factor 1 (IGF-1) has been shown effective in regulating tissue microenvironment and therefore survival and differentiation of stem cells in cardiac TE applications. Cardiac ECM proteins such as GSN, VDAC2, and HSPA11 are reported to regulate inhibition of cell death and control of actin filament activity in cell/scaffold constructs. The incorporation of cardiac-derived genes including Tmsb4× and Psap has shown to increase the rate of cell proliferation, adhesion, and migration in certain gelatin-based cardiac TE scaffolds [35].

The degradation profiles previously described for PLA, PLGA, and PCL are important for several reasons. Most importantly, the in vivo biodegradation rate of a TE scaffold’s material must match the rate of de novo tissue formation from the cells seeded onto it [36]. This is imperative, and if an appropriate biodegradation rate is not achieved, defects and cavities will form within the TE construct, leading the construct to fail. Of additional importance, the physical and chemical properties of a TE scaffold change during cell culture and tissue formation and growth. Therefore, the degradation profile of a TE scaffold must be rigorously studied to ensure that the scaffold does not deviate away from its physical and chemical requirements [37]. Methods of degradation rate control for TE scaffolds as described in a review by Zhang, Zhou, and Zhang in 2014 include scaffold material composition, microenvironment control, scaffold structure, surface treatment, external energy intervention, and physical loading [37].

The material composition of a polymeric scaffold is the primary factor that determines its hydrophilicity and its rate of degradation because the degradation rate of the bonds present in a polymer strongly affects the degradation rate of the overall polymer. The composition of polymer chains also affects the overall degradation rate. Blending and copolymerization are often employed to modify the degradation rates of polymeric scaffolds [37].

### 2.3. Physical and Mechanical Properties of TE Scaffolds

The microenvironment of a polymeric scaffold exerts many signals, cues, and chemical, biological, and mechanical forces onto it. Therefore, control of the microenvironment for a TE scaffold can strongly influence its degradation rate, profile, and behavior. Within the microenvironment, one of the most influential parameters is the pH value because it affects the rate of biodegradation [38]. For example, in vitro biodegradation of PLGA suggests that both alkaline and strongly acidic media accelerates degradation [39].

The structural properties of TE scaffolds have a high influence on degradation rates. One of the most influential structural properties is the surface-area-to-volume ratio; it has been shown that higher surface area ratios lead to higher matrix degradation. This relationship is particularly important in nano-scaled scaffolds [37].

Surface treatment and modification is one method of modifying degradation rates, profiles, and behaviors for TE scaffolds, particularly polymeric ones. Surface modifications include magnetron sputtering, ion irradiation, plasma polymerization, and plasma treatment. One mechanism of surface modification focuses on changing the hydrophobicity profile of a scaffold, i.e., increasing or decreasing its water intake to affect the hydrolysis process, and thereby increasing or decreasing the degradation rate and profile of the TE scaffold [37].

The term “weathering” refers to sources of external energy input that increase the degradation rate. Weathering sources include ultraviolet radiation, ultrasound, heat, and other environmental stresses, all of which increase the degradation rate of a polymeric TE scaffold and affect the degradation profile and behavior [37].

Finally, physical loading may be an influential factor for the degradation rate. A study by Li et al. in 2010 observed higher degradation rates of PLA and PLGA scaffolds when under tensile force. They also noted, however, that the degradation of other biodegradable scaffolds such as those comprised of PCL and chitosan, were not influenced by physical loading [40].

The fiber diameter and pore size of a TE scaffold are of utmost importance. The fiber diameter and pore size of nanofibrous scaffolds are directly related. If the fiber diameter and pore size of a TE scaffold is not in the appropriate range, it can lead to the scaffold functioning as a “sieve” and keeping cells on the scaffold’s surface, thus preventing cell migration into the interior of the scaffold. The pore sizes found in the scaffolds are vitally important because if the pore size is too small, it may interfere with cellular infiltration of the scaffold. Conversely, if the pore size is too large, it may impede cellular attachment to fibers. The minimal pore size for a TE scaffold should similar to the diameter of the cells desired to adhere to, migrate onto, and proliferate on the scaffold [41].

Structural and mechanical properties of TE scaffolds include both those desirable in a TE scaffold and those that stimulate and facilitate desirable responses from the cells seeded onto them. The structural and mechanical properties desirable vary based on the type of TE, i.e., bone, cardiac, neural, dental, muscle, liver, etc. This review is focused on cardiac TE, more specifically TE of the heart. For TE of the heart, the desirable structural and mechanical properties of scaffolds center upon the need to accommodate if not actively facilitate the heart’s electrical action potentials and contractile forces [5].

These factors of biocompatibility, in vivo degradation rate and profile, fiber diameter, pore size, surface-area-to-volume ratio, other surface properties, structural and mechanical properties, and other factors, all combine to determine the success or failure of a TE scaffold. Ultimately, a successful TE scaffold is one that offers appropriate biocompatibility and a degradation rate and profile that matches the rate of de novo tissue formation and has the fiber diameter, pore size, surface-area-to-volume ratio, surface properties, and structural and mechanical properties which are as biomimetic as possible and facilitate cellular attachment, proliferation, migration, differentiation, and maturation where each is appropriate.

## 3. Electrospinning

Electrospinning is an attractive fabrication method for cardiac TE scaffolds because it produces ultrafine fibers, ranging from the nano- to micro-scale in fiber diameter. Fibers of that scale demonstrate the optimal potential for mimicking the complex structure of the cardiac ECM [37,38,39,40] and have been proven effective in facilitating cardiac cellular proliferation and protein regulation [37,38].

**Figure 1:**
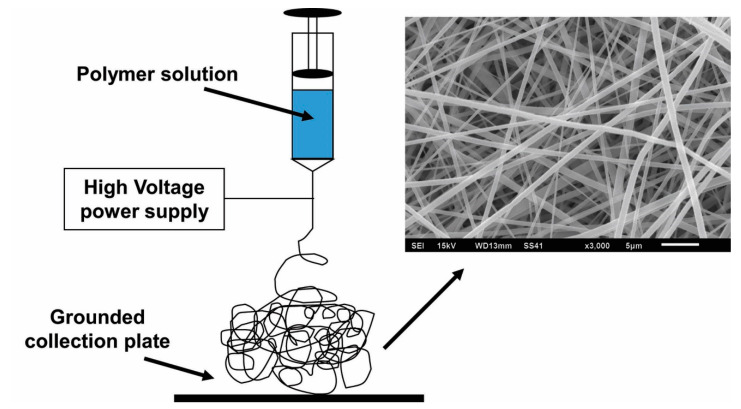
Depiction of the conventional electrospinning process.

The conventional electrospinning process, depicted in Figure 1 above, involves the extrusion of polymeric solution through a syringe and the application of a high voltage to the droplets that form. In the solution, the solvent must be conductive and highly volatile so that it will evaporate quickly as the solution is extruded. The electrostatic repulsion generated from the application of the electrical potential causes charge to accumulate at the surface of the polymer droplets as they emerge from the syringe tip. When the force of the electric field overpowers the cohesive force of the solution, a charged stream of the solution erupts from its surface. This point of eruption is referred to as a “Taylor cone.” As the stream dries in midflight from the syringe tip to the collection plate, elongation occurs due to electrostatic interactions between charges within the stream, while simultaneously the solvent evaporates and solidifies the stream into fibers, which are deposited onto the collection plate, forming a nonwoven mesh of randomly aligned nanofibers [17,26,42,43].

This is an attractive method for generating fibers used in TE scaffolds for many reasons. The fiber and pore sizes achievable through electrospinning are ideal for cardiac TE applications. The optimal range for cardiac TE scaffolds is a 10–100 μm pore size and 50–200 μm fiber diameter [41,42,44]. The ideal biomimicry of the myocardial ECM occurs when nanofibers have diameters around 500 nm [45]. Electrospinning typically generates fibers ranging from 20 nm to 20 μm in fiber diameter [46]. Fiber diameter has been shown to have the ability to regulate and direct cell behavior [47] as discussed in the previous section. Most notably, fiber structures in the nanoscale, which electrospun fibers typically are, tend to correlate with high surface-to-volume ratios, which provide a greater area for cell attachment, as previously discussed [29,41].

Scaffolds composed of electrospun fibers exhibit nano- and micro-scale structures that mimic the in vivo myocardial ECM, mainly through its architecture. An ECM microenvironment is traditionally described as having both a fibrillary protein structure and an amorphous matrix [12]. The electrospun fibrous network and anisotropy of the fibers mimic cardiac muscles’ hierarchical organization of the fibrillary protein structure found within the native cardiac ECM [5,12,15,16,17,18]. This biomimicry achievable through electrospun scaffolds is important because the structure of the ECM contributes to functional intercellular interactions that provide mechanical and biochemical support to surrounding cells, the nature of which depends on tissue type [40,41,42]. Therefore, if electrospun fibrous scaffolds are capable of structurally mimicking the ECM, then, in theory, this should allow for cardiac TE constructs using electrospun fibrous scaffolds to facilitate intercellular interactions similar to those occurring in vivo due to the ECM’s structure.

Though electrospun scaffolds offer much promise in biomimicry, achieving biomimetics for cardiac TE is a complicated endeavor, because the ECM of the heart is so complex. The cardiac ECM is hierarchically organized into epimysial, perimysial, and endomysial fibers, which together form a complex fibrous network [46]. Epimysial fibers surround the heart muscle and prevent it from stretching excessively [48]. They are larger, with fiber diameters on the scale of several micrometers. Perimysial fibers are smaller, averaging 1 μm diameters, and compose a mesh that surrounds cardiac muscle bundles and connects adjacent bundles, thus facilitating anisotropic contractions of the heart muscle [48,49]. Endomysial fibers, which vary in size from diameters of tens to hundreds of nanometers, form meshes, which envelop individual CMs and penetrate the cell plasma membrane to form interactions between the ECM and cytoskeleton proteins [48]. Together, these three types of fibers comprise a unique architecture that forces cardiac cells to establish cell–cell electrical and mechanical coupling, thus forming aligned cell bundles [46]. This organization of fibers gives rise to unique topographical features of cardiac ECM, to which the cells interacting with the ECM are highly sensitive [49]. Research by Bettinger et al. [50] and Li et al. [51] suggests that these ECM topographies influence cell morphology, proliferation, and differentiation even at the single-cell level.

Modifications to electrospinning have shown promise in acquiring scaffolds that mimic the complex hierarchy of native tissues. In 2018, Jun et al. [40] summarized the capabilities of different electrospinning techniques to fabricate nanoscale/microscale fibrous structures with interconnected pores resembling the highly complex, naturally occurring structures of in vivo ECM in tissues. They stressed that electrospun fibrous scaffolds with varying degrees of fiber alignment demonstrate an outstanding ability to guide cell morphology and function. Jun et al. outlined how geometrical control of fibrous scaffolds can be achieved through dual extrusion electrospinning, temperature-assisted electrospinning, micropatterned collectors for electrospinning, and post-processing of electrospun fibers, as well as how three-dimensional scaffolds can be acquired through liquid-collecting electrospinning, gas foaming, self-assembly, fibrous yarn scaffolds, hydrogel-integrated fibrous scaffolds, and near-field electrospinning with 3D printing technology [40]. Relevant to cardiac TE is the use of micropatterning to mimic the microstructure of the cardiac ECM [9] and the integration of hydrogels into fibrous scaffolds to facilitate cell migration and cellular contact guidance [48].

Electrospinning is also an attractive scaffold fabrication method for TE applications because of the many studies [17,40,42,45,49,52,53,54,55] relating solution and processing parameters to functionally relevant fiber and mesh properties. These solution parameters include polymer type and polymer molecular weight, solution concentration, solution viscosity, and solution conductivity, while the processing parameters include the magnitude of applied voltage, die-collector distance (DCD), collector type, and flow rate [41,42,52,53,54,56]. Elucidating the relationship between these electrospinning parameters and resultant properties of electrospun fibers (such as fiber diameter, pore size, and porosity) allows the achievement of fiber characteristics which are ideal in TE scaffolds for specific applications. For example, fiber diameter plays an important role in determining cell attachment and proliferation, because the diameter of the fibers dictates how well the cells can attach to them and how they can proliferate around them. In the electrospinning process, fiber diameter is dependent on solution viscosity, and can thus be varied by varying polymer type and concentration. In general, fiber diameter increases proportionally with polymer concentration [41,42]. This relationship allows for the tuning of fiber diameters to appropriate ranges for cell attachment and proliferation.

In addition to controlling fiber properties, electrospinning also offers a degree of control over mesh properties. Mesh configuration can be influenced by the manipulation of parameters such as fiber orientation, fiber positioning density, and fiber length [12]. Mesh porosity depends on solution properties, and fiber alignment can be controlled by employing different collector types, such as implementing rotating mandrels with controlled rotary speed to achieve varying degrees of alignment. These relationships between electrospinning parameters and mesh properties are important because they allow for tuning of electrospinning processes to produce meshes which mimic the structure of the cardiac ECM. Electrospinning offers the additional advantages of being a simple, cost-effective, and versatile fabrication process [41,42].

### Polymers and Fabrication of Electrospun TE Scaffolds

Materials used for electrospun TE scaffolds include synthetic and natural polymers, polymer blends and composites, and ceramics. The most commonly used electrospun polymers for cardiac TE are PCL, PLLA, PLGA, polyurethane (PU), and polyhydroxy butyrate (PHB), as well as various polymer blends and core-sheath polymer combinations of those listed [42,55]. Research using electrospinning to develop TE scaffolds is summarized in Table 1.

In 2017, Wang et al. developed an electrospun nanofibrous scaffold composed of a PLA and PANI blend [47]. They aimed to fabricate a PLA scaffold that structurally mimicked the ECM of the native myocardium and to incorporate PANI into the scaffold to obtain sufficient conductivity to promote electrical propagation for the functional coupling of CMs. By incorporating 0–3 wt% PANI into PLA, the resultant electrospun nanofibrous sheets demonstrated enhanced electrical conductivity without affecting fiber diameter, approximately 500 nm. The conductivity of the nanofibrous sheets increased with increasing PANI content, and the samples demonstrated conductivity values of 3.6 ± 0.7 × 10^−6^ S/m (PLA/PANI1.5) and 2.1 ± 0.3 × 10^−5^ S/m (PLA/PANI3 doped with camphor sulfonic acid), which are sufficient for the conduction of electrical signals in vivo. Additionally, the PLA/PANI nanofibrous sheets showed good cell viability and proliferation using H9c2 rat cardiomyoblasts and promoted differentiation of H9c2 cells in terms of myotube number, myotube length, maturation index, and fusion index. Furthermore, PLA/PANI nanofibrous sheets enhanced cell-cell interaction and maturation of primary CMs and promoted spontaneous beating. Wang et al. [47] also used the PLA/PANI nanofibrous sheets to form 3D bioactuators with tubular and folding shapes and found that spontaneous beating of CMs occurred at a higher frequency than for nanofibrous sheets of only PLA. In vivo, an individual in a resting state would have a lower beating frequency relative to an active state. Consequently, the tissue-engineered constructs should be capable of accommodating a range of beating frequencies to match the tissue demands in vivo. Additionally, the spontaneous beating achieved should propagate in a controlled fashion from the state of origin to the rest of the heart. This research suggests that specific modifications to electrospun scaffolds to introduce tunable electroconductivity, such as the incorporation of PANI, facilitate the development of electrophysical properties of CMs, and thereby advance the field of cardiac TE [47].

Hsiao et al. (2012) developed an electrospun mesh composed of aligned composite PANI and PLGA nanofibers doped with HCl [57]. They aimed to create an electroactive scaffold to coordinate the synchronous beating of CMs. Hsiao et al. found that doping the electrospun mesh with HCl imbued the mesh with conductive properties, giving it a positive charge, which attracted negative adhesive proteins, fibronectin, and laminin, thereby enhancing cell adhesion. Neonatal CMs isolated from Wistar rats adhered well to the meshes, forming isolated cell clusters. The cells within these clusters demonstrated morphological alignment along the major axis of the nanofibers within the mesh. Cell culture of the neonatal CMs revealed the expression of gap-junction protein connexin 43 and synchronous beating of CMs within each cluster. Hsiao et al. were able to synchronize this beating through the application of electrical stimulation: trains of electrical pulses at 1.25 Hz and 5 V/cm, which is characteristic of the native myocardium and therefore designed to mimic the electrical activity of a heart in vivo. The beating behavior of the CMs was then quantified through analyses of video recordings. This research by Hsiao et al. was a valuable contribution for myocardial TE, which requires the establishment of electrical integration within the scaffold and synchronous beating of CMs [57].

In 2005, Zong et al. explored fine-textured electrospun scaffolds for heart tissue constructs [58]. They cultured neonatal murine primary CMs onto electrospun PLGA scaffolds and found that the fine fiber architecture of the scaffolds allowed the CMs to form well-connected tissue. The scaffolds exhibited a large distribution of fiber diameters, averaging 1 µm. SEM revealed the development of sub-micron features to mimic the cardiac ECM was successful. These features included fine-fiber architecture which provided external cues for isotropic or anisotropic growth of CMs, which to some extent crawled inside and pulled on fibers. It also referred to the preference of CMs for relatively hydrophobic surfaces. Seeding with primary CMs from Sprague–Dawley rats was performed, found that these primary CMs developed into constructs that exhibited well-defined periodic units within sarcomeres. The constructs also exhibited cell-to-cell contacts that were comparable to those exhibited in well-connected adult tissue. Based on these two criteria, these constructs were classified as “tissue-like.” On the electrospun PLGA scaffolds, the CMs developed sarcomeres, i.e., mature contractile machinery. Electrical functionality, quantified by excitability, was confirmed with optical imaging of electrical activity using the voltage-sensitive dye di-8-ANEPPS. From this imaging, Zong et al. concluded the scaffolds provided the cells with appropriate electrochemical modulation [58].

In 2011, Orlova et al. published research exploring architectural control of electrospun nanofibers to address thickness limitations for CMs [12]. As previously discussed in this review, the oxygen requirements of CMs functionally limit engineered tissue thickness. Orlova et al. cited the critical limit for tissue-engineered cardiac constructs as <100 μm, due to restricted oxygen and nutrient flow via only passive diffusion. Orlova et al. focused on the architectural configuration of electrospun fibrous meshes, as controlled by varying positioning density and degree of alignment. Meshes composed of electrospun polymethylglutarimide (PGMI) nanofibers were either imprinted onto a glass coverslip or suspended in space across a polydimethylsiloxane (PDMS) holder in an attempt to maintain three-dimensionality and structural anisotropy of the scaffolds while facilitating a construct that was permeable to nutrients and metabolites while overcoming diffusion-based limitations on the thickness of cardiac TE constructs. Their fabrication method allowed them to obtain meshes with randomly or coaxially (i.e., concentric layers or “core-sheath”) oriented structures, differing fiber diameters, and various fiber positioning densities [12].

Orlova et al. [12] found that micro-imprintation on solid substrate nanofibers guaranteed aligned cell growth when the distance between the nanofibers was 30 μm or less. When suspended in 3D mesh, the orientation and positioning density of the nanofibers dictated the overall structure of the tissue. When the PGMI nanofibers were spaced with an average distance of 180–150 μm between them, there was little effect aside from minor guidance to the cells in their immediate vicinity, resulting in the cells acquiring a slightly elongated shape. When nanofiber spacing increased in density, with an average 100–120 μm distance between them, cells acquired a preferred orientation, assimilating towards the orientation of the nanofibers without any considerable elongation. This pattern continued as positioning density further increased to an average of 50–70 μm distance between nanofibers. In this configuration, cell alignment and elongation both increased. Finally, at the highest density of nanofiber spacing, when the distance between nanofibers was approximately 20 μm, the maximum level of cell ordering and elongation was observed, with cells well-aligned and their anisotropic growth in the direction of the nanofibers occurring as a result. Orlova et al. seeded the meshes with cardiac cells isolated from the ventricles of neonatal Wistar rats. The meshes facilitated the proliferation of the cardiac cells into contractile tissue filaments, open-worked tissue meshes, and continuous anisotropic cell sheets [12].

In 2019, Shokraei et al. aimed to develop an electroconductive scaffold composed of hybrid nanofibers created from simultaneous electrospinning and electrospraying of polyurethane/carbon nanotube (CNT) composites [59]. The inclusion of multi-walled CNTs was shown to increase the conductivity of the scaffold. These hybrid nanofibers demonstrated sufficient biocompatibility when seeded with H9c2 cells and human umbilical vein endothelial cells (HUVECs). The scaffolds also demonstrated high viability and proliferation of cardiomyoblasts, and improved interactions between the scaffold and cardiomyoblasts [59].

These studies demonstrate some of the recent research in developing electrospun scaffolds specifically for cardiac TE applications. They emphasize the capability of electrospun fibers to mimic natural structures of the cardiac ECM, the demonstration of sufficient cell viability on these electrospun scaffolds, and the necessity of appropriate electroconductive properties within a scaffold. Most notably, each study, through a different method, aims to biomimic the in vivo environments that CMs experience.

## 4. Induced Pluripotent Stem Cell-Derived Cardiomyocytes

In 2006, Takahashi and Yamanaka discovered that murine fibroblasts could be reprogrammed into an embryonic-like state by transduction of a specific set of transcription factors (Oct3/4, Sox2, c-Myc, and Klf4) [60]. The resultant cells, which Takahasi and Yamanaka called induced pluripotent stem cells (iPSCs), demonstrated the capacity to differentiate into cell derivatives of all three primary germ layers. In 2008, Mauritz et al. published research wherein these murine iPSCs were differentiated into functional murine CMs [61].

In 2007, Takahashi et al. translated their iPSC discovery to human cells, successfully deriving iPSCs from adult human fibroblasts using the same four transcription factors [62]. These human iPSCs exhibited morphology, proliferation, surface antigens, gene expression, epigenetic status of pluripotent cell-specific genes, and telomerase activity similar to that observed in human embryonic stem cells. Like their murine counterparts, these human iPSCs were capable of differentiating into derivatives of all three primary germ layers, and therefore potentially every type of cell present in the human body.

### 4.1. Cardiac Differentiation of iPSCs

The process of differentiating iPSCs into CMs is now understood and optimized. In 2012, Mummery et al. published a methods overview describing the basic biology of iPSC-CM differentiation and the available state-of-the-art protocols [63]. The in vitro differentiation of stem cells into CMs follows the same sequential stages as embryonic cardiac development, wherein three families of protein growth factors are thought to control early mesoderm formation and cardiogenesis: bone morphogenic proteins (BMPs), wingless/INT proteins (WNTs), and fibroblast growth factors (FGFs). After extensive study, it was concluded that the timeline and relative expression of these growth factors in a specific combination induces the cardiogenic mesoderm. Anteriorly migrated mesodermal cells, in response to the appropriate signals, switch on a heart-specific combination of transcription factors that establishes the cardiac transcriptional program. Most notably, T-box factor Brachyury T and Mix11 are expressed by mesodermal precursor cells in the primitive streak; Mesp1 is transiently activated as cells enter the “cardiac” mesoderm stage of development; a subset of the Mesp1+ population expresses Nkx2–5, Tbx5, and Is11, which are early markers of cardiac lineage activated during heart fields formation; Nkx2-5 and Tbx5 associate with GATA and SRF factors to activate cardiac structural genes; GATA4 regulates early heart tube formation and GATA6 facilitates myogenesis; and members of transcription family MEF2 regulate cardiac muscle structural genes. These multiple complex interactions between gene regulatory networks control the initial differentiation, proliferation, and maturation of CMs, and can be used as markers for human iPSC-CM cultures. Most protocols for inducing cardiomyogenesis in iPSCs operate through the activation or inhibition of these signaling pathways [63].

Although there are various genetic markers for cardiac progenitor cells (CPCs), there is no “gold standard” marker. This is because there are several populations of CPCs in the heart and there is an overlap in the expression of currently identified genetic markers between the CPCs. Some examples of CPCs include cardiosphere-derived cells, c-Kit^+^ CPCs, epicardium derived cells, cardiac side population cells, and cardiac colony-forming unit fibroblasts [63]. Some of the overlapping genetic markers associated with those CPCs include membrane markers (such as CD34, CD90, Flk-1/KDR, Sca-1, and Abcg-2) and transcription factors (such as Isl-1, MEF2C, and GATA4) [64]. As CPCs are located in all chambers of the heart, this adds a layer of complexity to determining the most appropriate genetic marker for identifying a specific type.

The three major approaches for differentiation of human iPSCs to CMs are embryoid body (EB)-mediated culture, monolayer culture, and inductive co-culture. Many earlier methods included maintaining undifferentiated iPSCs on feeder layers. EB-mediated culture, as the name suggests, uses EBs, which are three-dimensional aggregates of iPSCs. The first successful EB-mediated differentiation used human embryonic stem cells (hESCs), wherein collagenase IV was used to disperse hESCs colonies into small clumps of 3–20 cells [9]. These clumps were grown as suspension EBs in Petri dishes. This process was later modified to differentiate human iPSCs from CMs [9]. This suspension method and a hanging drop (HD) method (wherein equal numbers of cells are dispersed in gravity-induced, physically separated cell aggregates suspended from the lid of a petri dish) are the two current conventional methods of EB-mediated culture [65]. This method, however, has been criticized for its inefficiency, as the culture typically only led to a yield of 1% or less CMs, and the process produces variable results in different human pluripotent stem cell lines [66]. In monolayer culture, cells are directed into cardiac differentiation by the application of growth factors [63]. Innovations by Zhang et al. in 2010 incorporate ECM and cytokines into this process, increasing its reproducibility and the robustness of the CMs it generates [67]. Inductive co-culture refers to the differentiation of CMs from iPSCs by co-culturing them with visceral endotherm-like cells. This process is attractive for its conveniences, which include requiring few cells, progressing rapidly, having a simple execution, and producing a sufficient volume and quality of CMs for the detection of visible beating and identification of sarcomere structures by immunofluorescent staining [68].

It is widely debated whether EB or monolayer culture for iPSC-CMs is more effective and/or appropriate. Monolayer culture is a two-dimensional differentiation procedure, as opposed to using an EB formation approach, which is a three-dimensional procedure [69]. Monolayer differentiation offers higher differentiation efficiency than the EB approach, more reproducible differentiation, and higher susceptibility to growth factors in the ECM microenvironment [70]. Purification of the iPSC-CM population via sorting is easier to achieve in regards to the dissociation methods required to create a single-cell suspension. Purification of the CM population can be dependent upon the successful formation and subsequent dissociation of EBs. An alternative to the labor-intensive manual dissociation steps required for further purification is to genetically engineer iPSCs to include a fluorescent reporter protein under the control of a cardiac-specific genetic promoter [63]. However, it necessitates the optimization of growth factors and Matrigel concentration and presents significant genetic heterogeneity during maturation within the cultures it produces, if it is performed in high passages. Conversely, EB differentiation offers high EB homogeneity, consistency between runs, control of EB sizes by three-dimensional cell patterning systems, and controllable growth factor administration. It also does not require any specialized equipment [70].

In 2017, Jeziorowska et al. conducted experiments comparing the characteristics of iPSC-CMs on differentiation day 27 ± 2 using EB vs. monolayer culture with two different Wnt-inhibitors: IWR1 which inhibits Wnt response, and IWP2 which inhibits Wnt production [69]. They found that the level of Troponin T (TNNT2) expressed was significantly higher and the sarcomere length was greater for iPSC-CMs seeded in monolayer cultures vs. EB ones. They also found that sarcomere maturation was higher for monolayer protocols using IWP2 as opposed to all other procedures. Jeziorowska et al. concluded that monolayer culture with IWP2 allows the production of a higher yield of iPSC-CMs.

In 2017, Correia et al. compared monolayer to EB differentiation [71]. They found that EB differentiation displayed downregulation and increased expression of certain genes desirable for CM cultures. Most importantly, they demonstrated that EB differentiation reproducibly improved CM purity and metabolic maturation of CMs across different human PSC lines.

In 2012, Lian et al. published a protocol describing the successfully directed differentiation of CMs from human PSCs by the temporal modulation of Wnt and β-catenin signaling in a completely defined, serum-free system [72]. This system is a monolayer-based directed differentiation platform which sequentially exposes the human PSCs to Activin A and BMP4 in a defined RPMI/B-27 medium. It offers a much more efficient alternative to the EB-mediated differentiation of human PSCs described above, generating greater than 30% CMs in the H7 human embryonic stem cell line. The method offers another advantage in that it is one of the first methods which is xeno-free, making translation to clinical applications easier.

### 4.2. iPSC-CMs Used for Tissue Engineering

iSPCs herald in promising innovations for the field of cardiac TE because they theoretically offer the ability to create limitless numbers of CMs. However, a primary limitation of iPSC-CMs is that they are immature relative to functional adult CMs, demonstrating immature electrophysiology, morphology, mechanics, and metabolism. Primary adult CMs are morphologically rectangular and exhibit distinctive electrical and mechanical properties, which, despite the development of highly efficient iPSC-CM generation protocols, iPSC-CMs still lack. iPSC-CMs typically demonstrate amorphous or circular cell morphologies with disorganized sarcomeres and therefore underdeveloped sarcoplasmic reticulum and transverse tubule networks [6]. As a result, excitation-contraction (EC) coupling does not occur the way it does in vivo, which utilizes both intracellular calcium stored within the cardiac sarcoplasmic reticulum and extracellular calcium. Instead, contraction is mediated principally through extracellular calcium influx.

Overall, iPSC-CMs are typically so immature that they demonstrate electrical and mechanical properties similar to embryonic CMs, despite expressing many of the same proteins and having the same ionic currents as adult CMs [10]. This is highly problematic because if immature CMs were implanted into an adult heart, they would be incapable of electrically coupling with mature CMs to propagate the electrical signals across the heart to facilitate its beating [6]. Research aiming to improve iPSC-CM maturity includes developing long-term culture methods, altering culture media, implementing electrical and mechanical stimulation, and employing matrix patterning.

### 4.3. iPSC-CM Maturity for TE Applications 

Current studies evaluating iPSC-CM maturity so they can viably be used for cardiac TE applications is summarized in Table 2. In 2013, Lundy et al. published research indicating that long-term in vitro culture of human iPSCs and human embryonic stem cells (ESCs) resulted in morphological, contractile, electrophysiological, and genetic maturation [73]. Morphologically, late-stage (i.e., 80–120 days of culture) iPSC-CMs and ESC-CMs demonstrated increased cell size, anisotropy, myofibril density, sarcomere visibility, and the fraction of multinucleated CMs compared to early-stage (i.e., 20–40 days of culture) ones. Contractile performance, calcium handling, action potential amplitude, upstroke velocity, and induction of key cardiac structural genes all improved for late-stage iPSC-CMs and ESC-CMs when compared to early-stage ones. Lundy et al. [73] concluded that long-term in vitro culture is a viable method to contribute to the maturation of iPSC-CMs and ESC-CMs into cells that more closely phenotypically and genetically resemble functional adult CMs. Liaw et al. (2015) point out that the human gestational period is approximately 40 weeks, as opposed to the majority of human tissue-engineered cardiac tissue, which is typically cultured for 3–4 weeks [74]. This contextualizes Lundy et al.’s work in 2013 to assess if long-term culture increases the maturity of CMs [73].

Hirt et al. (2014) applied continuous electrical stimulation to cardiac TE constructs composed of neonatal rodent CMs or human iPSC-CMs in a culture medium [75]. They applied electrical stimulation to the constructs starting on the fourth day of culture. This electrical stimulation resulted in higher CM density and increased connexin-43 abundance. These results are desirable because Connexin-43 is an integral membrane building-block for the formation of gap junctions in the heart. These gap junctions directly transmit the depolarizing current cell-to-cell across the chambers of the heart, giving rise to its synchronous beating, which is electrically coupled across the CMs [76]. Furthermore, Hirt et al. observed a shift of the Ca^2+^ response curve towards more physiological values. This indicates that continuous pacing improved the structural and functional properties of the CMs in the TE construct [75].

Liaw et al. (2015) took a mechanical rather than electrical approach to the maturation of CMs, attempting to recreate the pumping action of the human heart, which requires considerable mechanical force to compress the blood-filled chambers [74]. The heart’s rate of contraction is dictated by the spontaneously active sinus node and its transmission of electrical impulses into the ventricle. Through the systematic testing of different loading conditions to facilitate isometric contractions, isotonic contractions, and auxotonic contractions, Liaw et al. found that flexible loading was superior to other loading regimes. This was concluded by comparing flexible to static loading for isometric contractions, phasic loading for isotonic contradictions, and spring-based loading for auxotonic contractions. Liaw et al. used neonatal CMs for this research [74].

This research [73,74,75] demonstrates three of many approaches to maturing CMs for cardiac TE applications. Lundy et al. [73], Hirt et al. [75], and Liaw et al. [74] approach this goal from a cell culture, electrical, and mechanical perspective, respectively. Other approaches to mature iPSC-CMs include culture media alterations, physiological stimulation, matrix patterning, and altering metabolic and morphologic characteristics [10].

## 5. Electrospun Scaffolds Seeded with iPSC-CMs

Recent studies combining electrospun scaffolds with iPSC-CMs for cardiac TE are discussed in this section and summarized in Table 3. Most of these studies explore the effect of fiber anisotropy within the ES scaffold on the morphology, function, and behavior of iPSC-CMs seeded onto them [76,77,78,79]. Others explore the use of electrospun scaffolds seeded with iPSC-CMs in the treatment of dilated cardiomyopathy [80] and investigate the effects of the electrospun scaffolds’ polymeric compositions on the maturation of the seeded iPSC-CMs [81].

In 2016, Joanne et al. sought to use electrospun collagen scaffolds seeded with human iPSC-CMs to induce cardiac remodeling for the treatment of dilated cardiomyopathy [80]. They fabricated crosslinked collagen scaffolds using electrospinning and post-treatment crosslinking. They differentiated human episomal iPSCs into CMs, which were sutured into the ventricles of mice with or without implantation of the collagen scaffolds. Cardiac function and gene expression were then assessed. Joanne et al. found that the collagen scaffolds demonstrated sufficient biocompatibility both in vivo (as assessed in the aforementioned grafting experiments) and in vitro (as assessed in neonatal rat CMs seeded onto the scaffolds). They found that the presence of the collagen scaffold improved the cardiac function of the human iPSC-CMs when epicardially delivered to the mice. This improvement was associated with a statistically significant decrease in β-MHC expression and an increase in cardiac actin expression. Mice treated with the iPSCM-CM-loaded collagen scaffolds also demonstrated a significant increase in SRF gene expression, indicating improved scaffold vascularization induced by the iPSC-CMs. Joanne et al. hypothesized that closer contact between the host myocardium and the iPSC-CM-seeded collagen scaffold allowed the seeded iPSC-CMs to control the body’s inflammatory response to the implanted construct more effectively. Overall, Joanne et al. concluded that epicardial delivery of collagen scaffolds seeded with human iPSC-CMs improved functional outcomes of dilated mouse hearts [80].

Li et al. (2016) developed cardiac TE constructs comprising scaffolds of aligned, electrospun PMGI fibers and patterned human iPSC-CMs seeded onto them [77]. They used the extracellular recording to observe the activity of these TE constructs. The recordings revealed iPSC-CMs orienting anisotropically along the alignment of the electrospun fibers. They also showed premature CM beating, higher signal amplitude, and higher T-wave detection probability for aligned scaffolds compared to randomly oriented ones. Additionally, the recordings showed that iPSC-CMs seeded on the aligned scaffolds demonstrated anisotropic propagation of the field potential, indicating the formation of tissue-like constructs of matured CMs. These results signify the value of extracellular recording in the evaluation of TE constructs and the viability of TE constructs composed of anisotropically aligned electrospun scaffolds and patterned human iPSC-CMs [77].

In 2017, Wanjare et al. published research using anisotropic, microfibrous scaffolds composed of electrospun PCL to enhance iPSC-CM’s functional and organizational biomimicry of native in vivo CMs [78]. They electrospun PCL scaffolds with random orientation and 14 µm fiber diameter, and with parallel fiber alignment and 7 µm fiber diameter. Co-seeding both types of scaffolds with human iPSC-CMs and iPSC-derived epithelial cells (iPSC-ECs) revealed that anisotropic alignment of microfibers induced iPSC-CMs to orient along the fibers, promoted iPSC-CM maturation by increasing sarcomeric length and gene expression of myosin heavy chain adult isoform (MYH7), and increased maximum contraction velocity of iPSC-CMs. These results indicate that for cardiac TE applications, scaffold anisotropy plays an important role in the maintenance of iPSC-CM organization and contractile function [78].

In 2016, Han et al. [79] published research in seeming contradiction to that of Li et al. in 2016 [77] and Wanjare et al. in 2017 [78]. Han et al. compared maturation of human iPSC-CMs cultured on anisotropically vs. isotropically aligned PCL fibrous scaffolds. They cultured iPSC-CMs on tissue culture polystyrenes (TCPs) as a control. The iPSC-CMs exhibited anisotropy on anisotropic scaffolds and isotropy on isotropic scaffolds, as the authors predicted. iPSC-CMs cultured on anisotropic scaffolds exhibited higher expression of genes encoding the quantity of sarcomere proteins, calcium handling proteins, and ion channels. However, their calcium transient kinetics were slower than those of the iPSC-CMs cultured on TCPs. The higher the cycling rate for these calcium transient kinetics, the higher the frequency of CM contraction. Therefore, these results indicate that iPSC-CMs cultured on TCPs are more mature than those cultured on anisotropic scaffolds [79]. In this aspect, Han et al.’s research challenges the conclusions drawn by Li et al. [77] and Wanjare et al. [78].

Khan et al. (2015) compared morphological and functional changes in human iPSC-CMs seeded on highly aligned electrospun nanofibrous PLGA scaffolds vs. standard flat cell culture plates [76]. iPSC-CMs cultured on the aligned PLGA nanofibers demonstrated symmetrical alignment in the same direction as the PLGA nanofibers. Conversely, iPSC-CMs cultured on the plates exhibited random organization. The iPSC-CMs cultured on aligned fibers demonstrated more rapid calcium cycling (i.e., calcium transient kinetics) than those cultured on the plates. iPSC-CMs cultured on both surfaces robustly expressed the cardiac genes α-actinin, Troponin-T, and Connexin-43 in vitro, but only iPSC-CMs cultured on the aligned PLGA nanofibers displayed arrangement of cellular and gap proteins similar to that of normal in vivo cardiac tissue. Khan et al. concluded that the expressed phenotype of iPSC-CMs cultured on aligned nanofibers biomimic mature, native, in vivo CMs more closely than those cultured on standard cell culture plates [76].

Chun et al. (2015) electrospun scaffolds from combinatorial polymers comprising varying percentage molar ratios of the functional subunits PCL, polyethylene glycol (PEG), and carboxylated PCL (cPCL) [81]. They chose these monomers for specific properties relating to biodegradability, hydrophobicity or hydrophilicity, biocompatibility, and negative or positive charge. Using copolymers for the scaffolds allows each constituent monomer to introduce its characteristics, modifying the scaffold towards characteristics more ideal for cardiac TE applications. Copolymeric scaffolds were electrospun from 100% PCL, 90% PCL-10% cPCL, 4% PEG–96% PCL, and 4% PEG-86% PCL–10% cPCL, and a glass matrix was used as the control. Chun et al. found that iPSC-CMs cultured onto the 4% PEG–96% PCL scaffolds exhibited the greatest contractility and mitochondrial function. They also exhibited increased expression of the genes encoding intermediate filaments that transduce integrin-mediated mechanical signals to CM’s myofilaments. Finally, the iPSC-CMs cultured onto the 4% PEG–96% PCL scaffolds demonstrated a switch in the expression of the protein troponin I (TnI) from the fetal slow skeletal TnI (ssTnI) isoform, which in vivo is expressed gestationally and neonatally, to the cardiac TnI (cTnI) isoform, which in vivo is expressed in adults [82,83]. This is significant because the development of contractile properties within the human fetal heart are hypothesized to occur due to a complex combination of isoform changes such as that from ssTnI to cTnI. This isoform change also correlates to the development of myofilaments [82]. Because cTnI is only expressed in adult CMs, Bedada et al. proposed the isoform change from ssTnI to cTnI as a marker for adult maturation of cardiac tissue [83]. This research by Chun et al. in 2015 [81] contains the first reported switch from ssTn1 to cTn1 observed in vitro, and is hypothesized to indicate the same CM maturation that it signifies in vivo [83,84]. From their results, Chun et al. concluded that the 4% PEG–96% PCL scaffolds facilitated the induction of mechanotransductory pathways that activate maturation of iPSC-CMs [81].

Most of the recent research using electrospun scaffolds and iPSC-CMs for cardiac TE applications focuses on elucidating the relationship between scaffold anisotropy and cellular response-sometimes with conflicting conclusions [76,77,78,79]. Other research aims to determine ideal polymeric compositions for scaffolds [81] or use rehabilitative TE constructs to address cardiomyopathies [80]. The six studies discussed in this section, though far from exhaustive, represent the current state of knowledge regarding iPSC-CM-seeded electrospun scaffolds for cardiac TE applications. The contradictory conclusions drawn by Han et al. [79] as opposed to Li et al. [77] and Wanjare et al. [78] regarding the effects of scaffold anisotropy on iPSC-CM maturation are indicative of a relatively new field that is constantly progressing and requires much more research to reach stronger conclusions and consensuses.

## 6. Conclusions

Cardiac TE is a field that aims to repair, reconstruct, and replace cardiovascular structures, most notably the heart, with engineered tissues. This review briefly summarizes the components and challenges of cardiac TE, including iPSC-CM maturation, scaffold biomimicry and biocompatibility, and the body’s immune/host response to implanted TE constructs. Recent work demonstrates the ability to use electrospun scaffolds for cardiac TE purposes and various approaches to the maturation of iPSC-CMs. Methods of combining a biomaterials approach in the development of electrospun scaffolds seeded with iPSC-CMs for cardiac TE applications are boundless and offer promise for the direction of the field. If research in the field continues on its current course, cardiac TE will offer a strong and viable alternative to the current standard of care for cardiovascular disease and heart failure, ultimately alleviating much suffering and saving many lives.

## Figures and Tables

**Table 1:** Recent studies using electrospun scaffolds for tissue engineering (TE) applications [12,47,57,58,59].

Publication	Material	Fabrication	Cells	Requirement/Characteristics	Outcome
Wang et al. (2017) [47]	PLA/PANI blend	Electrospinning	H9c2 rat cardiomyoblasts	Incorporate PANI into electrospun PLA scaffolds to promote electrical propagation for functional coupling of CMs	Promoted differentiation into CMs; enhancement of cell-cell signaling and maturation of CMs; promotion of spontaneous beating within CMs
Hsiao et al. (2012) [57]	Composite PLGA and PANI doped with HCl	Electrospinning	Neonatal CMs from Lewis rats	Create electrospun mesh that serves as an electrically active scaffold to coordinate the synchronous beating of CMs, thus mimicking electroconductive properties of cardiac ECM	All CMs within each cluster demonstrated synchronous beating, implying fully developed electrical coupling between cells; beating rates within isolated CM cell culture could be synchronized via electrical stimulation designed to mimic human heart
Zong et al. (2005) [58]	PLGA	Electrospinning	Primary CMs from Sprague Dawley rats	Develop sub-micron features within electrospun meshes to mimic cardiac ECM	SEM revealed the development of sub-micron features successful; primary CMs cultured on electrospun scaffolds developed into tissue-like constructs; scaffold provided appropriate electrochemical modulation
Orlova et al. (2011) [12]	PMGI, some suspended on PDMS	Electrospinning	Cardiac cells from Wistar rats	Address tissue thickness limitations for engineered cardiac constructs via varying architectural configuration of electrospun scaffolds	Different architectural configurations in electrospun meshes achieved by varying positioning density and degree of alignment; cardiac cells proliferated into contractile tissue filaments, open-worked tissue meshes, and continuous anisotropic cell sheets
Shokraei et al. (2019) [59]	Poly-urethane with multi-walled carbon nanotubes	Electrospinning + electrospraying	H9c2 cells and human umbilical vein endothelial cells (HUVECs)	Use a simultaneous electrospinning + electrospraying method to create electroconductive nanofibrous patches that biomimic cell-cell communication capacity of the human heart in vivo	The increased conductivity of scaffold; high viability and proliferation of cells with increased cell/scaffold interactions

PLA = polylactic acid, PANI = polyaniline, CM = cardiomyocyte, PLGA = poly(lactic-co-glycolic acid), ECM = extracellular matrix, PMGI = polydimethylglutarimide, PDMS = polydimethylsiloxane.

**Table 2:** Recent studies aiming to improve iPSC-CM maturity [73,74,75].

Publication	Cells Used	Maturation Method	Results/Conclusion
Lundy et al. (2013) [73]	Human iPSC-CMs and ESC-CMs	Long-term culture to facilitate morphological, contractile, and electrophysiological maturation	Late-stage (i.e., cultured for longer) iPSC-CMs and ESC-CMs demonstrated higher morphological, contractile, electrophysical, and genetic maturity
Hirt et al. (2014) [75]	Neonatal rodent CMs or human iPSC-CMs	Application of continuous electrical stimulation	Higher CM density, increased connexin-43 abundance, and shift of Ca^2+^ response curve towards physiological values
Liaw et al. (2015) [74]	Neonatal rodent CMs	Mechanical recreation of pumping action in the human heart	Flexible loading most effective for the facilitation of contractions

iPSC-CM = induced pluripotent stem cell-derived cardiomyocyte, ESC-CM = embryonic stem cell-derived cardiomyocyte, CM = cardiomyocyte.

**Table 3:** Recent studies involving iPSC-CMs and electrospun scaffolds [76,77,78,79,80,81].

Publication	Electrospun Scaffold Material	Aim	Outcome
Joanne et al. (2016) [80]	Collagen	Use ES collagen scaffolds to deliver iPSC-CMs to the heart to induce cardiac remodeling in dilated cardiomyopathy	Collagen scaffolds exhibited high biocompatibility; iPSC-CMs delivered by ES scaffolds demonstrated improved cardiac function, scaffold vascularization, and scaffold adherence
Li et al. (2016) [77]	PMGI	Observe the activity of patterned human iPSC-CMs on aligned ES PMGI fibers through extracellular recording	Recordings showed iPSC-CMs organized into mature tissues oriented anisotropically along aligned ES fibers; recordings showed premature CM beating, higher signal amplitude, and higher T-wave detection probability compared to iPSC-CMs on non-aligned fibers; recordings showed that iPSC-CMs on aligned scaffolds exhibited anisotropic field potential propagation
Wanjare et al. (2017) [78]	PCL	Mimic highly ordered physiology and function of native CMs using anisotropic, microfibrous, ES PCL scaffolds seeded with human iPSC-CMs; compare the cellular response to anisotropic scaffolds vs. randomly oriented scaffolds	ES scaffolds with anisotropically aligned microfibers induced iPSC-CM alignment 2 days post-seeding, and promoted greater iPSC-CM maturation and higher maximum contraction velocity of iPSC-CMs, compared to ES scaffolds with randomly oriented fibers
Han et al. (2016) [79]	PCL	Seed human iPSC-CMs onto ES PCL scaffolds, using the anisotropic alignment of the PCL fibers to facilitate human iPSC-CMs’ mimicry of the longitudinal alignment into parallel bundles exhibited by CMs in in vivo adult myocardium	Cell alignment alone is insufficient to facilitate increased maturation in iPSC-CMs, based on the assessment of various gene expressions
Khan et al. (2015) [76]	PLGA	Compare morphological and functional changes in human iPSC-CMs cultured on highly-aligned, nanofibrous ES PLGA scaffold vs. standard flat culture plate	iPSC-CMs aligned symmetrically to ES PLGA fibers and demonstrated more rapid calcium cycling than CMs cultured on a flat plate; CMs expressed α-actinin, TnT, and Cx43 in vitro
Chun et al. (2015) [81]	Combinatorial polymer of PCL, PEG, and cPCL	Use ES combinatorial polymer matrices to facilitate in vitro maturation of iPSC-CMs	iPSC-CMs cultured onto 4%PEG-96%PCL exhibited the greatest contractility and mitochondrial function, TnI isoform switch from fetal ssTNI to postnatal cTNI, and increased expression of genes encoding intermediate filaments that transduce integrin-mediated mechanical signals to microfilaments

ES = electrospun, iPSC-CM = induced pluripotent stem cell-derived cardiomyocyte, PCL = polycaprolactone, PEG = polyethylene glycol, cPCL = carboxylated polycaprolactone, PLGA = polylactide-co-glycolide, PMGI = polydimethylglutarimide, TnI = troponin I, ssTNI= slow skeletal troponin I, cTnI = cardiac troponin I, TnT = troponin T, Cx43 = connexin 43.
